# REDUCING CARE REFUSAL IN OLDER ADULTS WITH DEMENTIA THROUGH HUMANITUDE CAREGIVER TRAINING

**DOI:** 10.1093/geroni/igae098.3083

**Published:** 2024-12-31

**Authors:** João Pärtel Araújo, Catherine Van Son, Helena Luz, Rosa Melo

**Affiliations:** Coimbra University, Aljubarrota, Coimbra, Portugal; Washington State University, MARYSVILLE, Washington, United States; University of Coimbra, Coimbra, Coimbra, Portugal; Escola Superior de Enfermagem de Coimbra, Coimbra, Coimbra, Portugal

## Abstract

**Background:**

Care refusal by older adults with cognitive impairment creates significant challenges for professional caregivers. Humanitude is a multimodal care method that promotes well-being during care for both care recipients and caregivers. This study evaluated the impact of Humanitude training for professional caregivers on refusal of care behaviors in older adults with cognitive impairment in an acute care setting.

**Methods:**

Two 4-day Humanitude care method (HCM) trainings were conducted for 23 healthcare professionals from six geriatric wards of an acute hospital. Training included theory, simulation, and clinical practice of Humanitude techniques with identified older adults. The participants identified 17 older adults with cognitive impairment who routinely refuse care. Refusal of care was measured at baseline and after Humanitude training using the Refusal of Care Informant Scale (RoCIS).

**Results:**

Most of the 23 healthcare professionals participating in the training were nurses, 78%, with an average of seven years of experience. The older adults identified were women (65%), with an average age of 83. All the older adults identified had a diagnosis of a major neurocognitive disorder, and the average Functional Assessment Staging Tool score was 4.9. The average RoCIS scores decreased by 71.4% after the participants used Humanitude techniques during the training’s clinical practice.

**Conclusion:**

A brief 4-day HCM training reduced care refusal by older adults with cognitive impairment. Implementing Humanitude care methods could improve caregiver capacity to address care refusal challenges and mitigate its effects on caregiving. Studies on Humanitude’s sustainability and outcomes are needed for caregiver and organizational adoption.

